# Viral vector delivery of Env trimer immunogens

**DOI:** 10.1186/1742-4690-9-S2-P341

**Published:** 2012-09-13

**Authors:** CL Parks, S Rabinovich, PJ Tiberio, KJ Wright, M Yuan, MG Delboy, M Kemelman, AJ Wilson, RL Powell, S Hoffenberg, MJ Chiuchiolo, C Boggiano, G Morrow, IC Lorenz, CK Jurgens, X Zhang, RW Lindsay, WC Koff, CR King, MJ Caulfield

**Affiliations:** 1 International AIDS Vaccine Initiative, Brooklyn, NY, USA

## Background

Our objective is to develop viral vaccine vectors that will elicit neutralizing antibodies that are specific for the functional attachment protein on the HIV particle. To achieve this goal, we are developing vectors that express membrane-anchored Env trimers that closely mimic authentic functional glycoprotein spikes.

## Methods

We are using vesicular stomatitis virus (VSV) as a vector platform for delivery of Env immunogens as transmembrane glycoproteins. We have investigated a variety of vector designs and Env modifications to identify combinations that balance the practical requirement for vector genetic stability with factors influencing antibody responses including immunogen abundance, efficient post-translational processing, and presentation of antigenic determinants representative of a functional trimeric spike.

## Results

Substituting domains in Env with analogous regions from VSV G, we have developed a number of immunogens that are efficiently expressed and incorporated in the infected cell plasma membrane, and in most cases, progeny virus particles. Antigenicity was evaluated using a panel of monoclonal antibodies specific for various Env epitopes.

## Conclusion

We identified modified Env immunogens that contain determinants for most classes of known broadly neutralizing monoclonal antibodies including those with specificity for the CD4 binding site (b12, PGV04), V3 and carbohydrate (PGT126), the MPER (2F5 and 4E10), the glycan shield (2G12), and structures formed by V1/V2 and carbohydrate (PG9, PG16, PGT145). Results from ongoing immunogenicity studies with vectors encoding SIV or HIV Env immunogens (subtypes A, B, or C) indicate that the modified trimers elicit antibody responses in small animals and nonhuman primates, and that some live vectors induce mucosal antibodies. Study sera are being analyzed for virus neutralization activity and fine specificity.

